# Susceptibility of subregions of prefrontal cortex and corpus callosum to damage by high-dose oxytocin-induced labor in male neonatal mice

**DOI:** 10.1371/journal.pone.0256693

**Published:** 2021-08-26

**Authors:** Eri Kitamura, Masato Koike, Takashi Hirayama, Takehiko Sunabori, Hiroshi Kameda, Hiroyuki Hioki, Satoru Takeda, Atsuo Itakura

**Affiliations:** 1 Department of Cell Biology and Neuroscience, Juntendo University Graduate School of Medicine, Tokyo, Japan; 2 Department of Obstetrics and Gynecology, Juntendo University Faculty of Medicine, Tokyo, Japan; 3 Advanced Research Institute for Health Science, Juntendo University, Tokyo, Japan; 4 Department of Neuroanatomy, Juntendo University Graduate School of Medicine, Tokyo, Japan; Rutgers University, UNITED STATES

## Abstract

Induction and augmentation of labor is one of the most common obstetrical interventions. However, this intervention is not free of risks and could cause adverse events, such as hyperactive uterine contraction, uterine rupture, and amniotic-fluid embolism. Our previous study using a new animal model showed that labor induced with high-dose oxytocin (OXT) in pregnant mice resulted in massive cell death in selective brain regions, specifically in male offspring. The affected brain regions included the prefrontal cortex (PFC), but a detailed study in the PFC subregions has not been performed. In this study, we induced labor in mice using high-dose OXT and investigated neonatal brain damage in detail in the PFC using light and electron microscopy. We found that TUNEL-positive or pyknotic nuclei and Iba-1-positive microglial cells were detected more abundantly in infralimbic (IL) and prelimbic (PL) cortex of the ventromedial PFC (vmPFC) in male pups delivered by OXT-induced labor than in the control male pups. These Iba-1-positive microglial cells were engulfing dying cells. Additionally, we also noticed that in the forceps minor (FMI) of the corpus callosum (CC), the number of TUNEL-positive or pyknotic nuclei and Iba-1-positive microglial cells were largely increased and Iba-1-positive microglial cells phagocytosed massive dying cells in male pups delivered by high-dose OXT-induced labor. In conclusion, IL and PL of the vmPFC and FMI of the CC, were susceptible to brain damage in male neonates after high-dose OXT-induced labor.

## Introduction

Induction and augmentation of labor is one of the most common obstetrical interventions all over the world [1, 2]. Fetal factors such as prolonged pregnancy and fetal growth restriction, and maternal factors such as hypotonic uterine dysfunction, premature rupture of membranes and preeclampsia are medically indicated [3, 4]. However, this intervention is not free of risks, although the benefits of labor induction typically outweigh the risks. For example, uterine contraction drug has a risk of causing adverse events, such as hyperactive uterine contraction, uterine rupture [5], and amniotic-fluid embolism [6]. Furthermore, according to the recent epidemiologic studies, the incidence of several mental disorders in offspring has been considered as one of the later risks of induction and augmentation of labor [7–9]. To reveal mechanistic insight into potential brain damage due to induced labor, we established a new animal model of induced labor with high-dose oxytocin (OXT) [10]. Of the three medications recommended for labor induction or augmentation (OXT, Prostaglandin F2α and Prostaglandin E2), OXT is the most commonly used to increase uterine contractions [3]. Our previous study [10] showed induced labor with high-dose OXT in pregnant mice resulted in male offspring-specific cell death in several specific brain regions, such as the prefrontal cortex (PFC), habenular nucleus and periventricular nucleus. In addition, the study reported the cell death was caused by OXT-induced uterine contraction and was not a pharmacological side effect of OXT itself. OXT administration to OXT receptor-knockout pregnant mice did not induce early delivery and brain damage in the pups. Furthermore, OXT administration to Wild-type pregnant mice did not directly influence OXT or OXT receptor expression in the brains of male offspring, or the numbers of OXT neurons in the brains of male adult mice [10].

Although our previous study using the new animal model of induced labor with OXT suggested increased cell death in the PFC [10], we had not analyzed brain damage in PFC subregions. The PFC plays a role in the regulation of three major aspects of social cognition: social motivation, social memory and social hierarchy, and is associated with mental disorders in both humans and rodents [11]. Therefore, in the present study we aimed to perform morphological investigation of the brain damage in detail in the PFC in male neonates born from the induced labor model mice established by our previous study [10].

## Materials and methods

### Animals

Wild-type C57BL/6J pregnant mice were purchased from CLEA Japan, INC. (Tokyo, Japan). The pregnant mice were housed one per one cage under a 12-h light/dark cycle and received food and water *ad libitum*. All animal experiments were carried out based on the recommended protocol of Juntendo University, and were approved after evaluation by the animal experiments committee of Juntendo University (Protocol Number: 1354). All efforts were made to minimize animal suffering and the number of animals used.

### Induced labor model

The induced labor model was established as previously described [10] with minor modifications. Briefly, on gestational day 18.5, mice were anesthetized using 4% and 2.5% isoflurane delivered with 0.4 l/min air for introduction and maintenance, respevtively. An osmotic pump (micro-osmotic pump, model 1003D, Alzet, CA, USA) was implanted subcutaneously in the anesthetized mice. Before the surgery, the pumps were filled with OXT acetate salt (BACHEM, Swiss) dissolved with phosphate-buffered saline (PBS) at a concentration of 6 μg/day (OXT group). Pumps filled with PBS were used for a control group (PBS group) and no pumps were implanted for the Wild group. The time until labor was defined as the time from implanting a pump to delivery. The time until labor of the Wild group was defined from 9:00 on gestational day 18.5 to delivery. In the OXT and PBS groups, micro-osmotic pumps were subcutaneously implanted into the pregnant mice at around 9:00 on gestational day 18.5. The time until labor was analyzed for each group, where n = 6–10 mother mice per group. The P1 survival rate was defined as the survival numbers at 24 h after birth per numbers of birth, and was counted in n = 2–5 mother mice per group. P1 body weight was defined as the weight at 24 h after birth, and was measured in n = 13–15 pups per group. We euthanized the remaining mice unnecessary for the current study by cervical dislocation.

### Sampling for histochemical analyses

The male pups in each group were analyzed at 24 h after birth for Nissl staining, terminal deoxynucleotidyl transferase dUTP nick end labeling (TUNEL), immuhohistochemistry and electron microscopy, and at around postnatal day 40 (P40) for Nissl staining. To induce hypothermia as anesthesia, they were immersed up to the neck in crushed ice and water and transcardially perfused with 4% paraformaldehyde with 0.1 M phosphate buffer (PB; pH 7.2; Fujifilm Wako, Osaka, Japan or Nacalai Tesque, Kyoto, Japan). The brains were dissected out and postfixed in the same fixative overnight at 4°C and embedded in paraffin. Coronal sections at 4-μm thick were cut with a microtome (SM2000R or RM2245; Leica Microsystems, Nussloch, Germany).

### TUNEL staining

TUNEL staining was performed using the DeadEnd colorimetric TUNEL system (Promega, Tokyo, Japan) as previously described [12]. After 3,3′-diaminobenzidine (DAB) staining was used for visualization and detection of TUNEL positive cells, Nissl staining was performed and samples were observed using an optical microscope (BX-51; Olympus, Tokyo, Japan). Additionally, when streptavidin Alexa Fluor^TM^ 594 conjugate streptavidin (1:200; Life Technologies, NY, USA) was used for visualization and detection of TUNEL-positive cells, samples were counterstained for 10 min with 50 ng/ml of 4′,6-diamidino-2-phenylindole dihydrochloride (DAPI) (Life Technologies, Carlsbad, CA) in PBS and observed using an inverted fluorescence microscope (BZ-X710; Keyence, Tokyo Japan) or a standard light microscope (BX51; Olympus) equipped with a DP72 digital imaging system (Olympus).

### Cell counting

TUNEL- and Nissl-stained brain sections and Iba-1-stained sections corresponding to Figure 61 of the Atlas of the Developing Mouse Brain at E17.5, P0, and P6 [13] were used for quantification of dying cells and microglia, respectively. One coronal section per mouse was chosen and brain regions of interest at 40× magnification were analyzed (one sample per mouse, n = 3–6 mice per group). Brain regions were defined according to the atlas [13]. The numbers of cells and nuclei were counted using the open source platform software Fiji [14], an image processing package and a distribution of ImageJ focused on scientific image analysis [15].

### Immunohistochemistry

Immunohistochemistry was performed as previously described with minor modifications [16]. The deparaffinized sections were treated with 10 mM citrate buffer (pH 6.0) at 80°C for 30 min for antigen retrieval and subsequently with TNB blocking buffer containing 0.1 M Tris HCl (pH 7.5), 0.15 M NaCl, and 0.5% TSA blocking reagent (Perkin Elmer, Waltham, MA). Sections were then incubated overnight at 4°C with rabbit anti-Iba1 (1:200; Fujifilm Wako) diluted in the TNB blocking buffer. For immunofluorescent microscopy, the sections were incubated at room temperature for 1 h with Alexa Fluor 488-conjugated goat anti-rabbit immunoglobulin G (IgG) (H+L) (1:200; Life Technologies) diluted in the TNB blocking buffer. Samples were then counterstained for 10 min with 50 ng/ml DAPI in PBS and observed using an inverted fluorescence microscope (BZ-X710). For immunohistochemistry for brightfield microscopy, the sections were incubated at room temperature for 1 h with goat anti-rabbit IgG conjugated with horseradish peroxidase polymer (ImmPRESS; Vector Laboratories, Burlingame, CA) Staining for peroxidase was performed using 0.0125% DAB and 0.002% H_2_O_2_ in 0.05 M Tris-HCl buffer (pH 7.5) for 5 min. Nissl staining was subsequently performed for some sections. Sections were observed with a BX50 standard microscope equipped with a DP72 digital imaging system.

### Transmission electronic microscopy

Transmission electronic microscopy was performed as previously described with minor modifications [17]. Briefly, the male pups in each group were analyzed at 24 h after birth. To induce hypothermia as anesthesia, they were immersed up to the neck in crushed ice and water and transcardially perfused with 2% paraformaldehyde and 2% glutaraldehyde (Nacalai Tesque) in 0.1 M PB (pH 7.2). The brains were dissected out, postfixed in the same fixative overnight at 4°C and sectioned at 1 mm using a brain matrices (Brain Science Idea. Co., Ltd., Osaka, Japan). Brain slices were post fixed with 1% OsO_4_, dehydrated with a graded series of ethanol, and embedded in Epon812 (Okenshoji, Tokyo, Japan). Semithin and ultrathin sections were cut with an ultramicrotome UC6 (Leica Microsystems, Vienna, Austria). Semithin sections were stained with tolidine blue and observed with an optical microscope (BX-51). Ultrathin sections were stained with uranyl acetate and lead citrate and examined with a transmission electron microscope HT7700 (Hitachi, Tokyo, Japan).

### Statistics

Variables were summarized using mean ± SE. Samples were classified into three groups; OXT, PBS (control), and Wild groups. Variables were compared between the two groups using Student’s *t*-test and between three groups using the Tukey–Kramer method or Steel–Dwass test (if the variables were not normally distributed or had unequal variances). A P value <0.05 was considered statistically significant. Statistical analyses were performed using EZR (Saitama Medical Center, Jichi Medical University, Saitama, Japan), which is a graphical user interface for R (The R Foundation for Statistical Computing, Vienna, Austria). To be exact, it is a modified version of R commander designed to add statistical functions frequently used in biostatistics [18].

## Results

### Verification of reproducibility of induced labor mouse model with OXT

**Fig 1:**
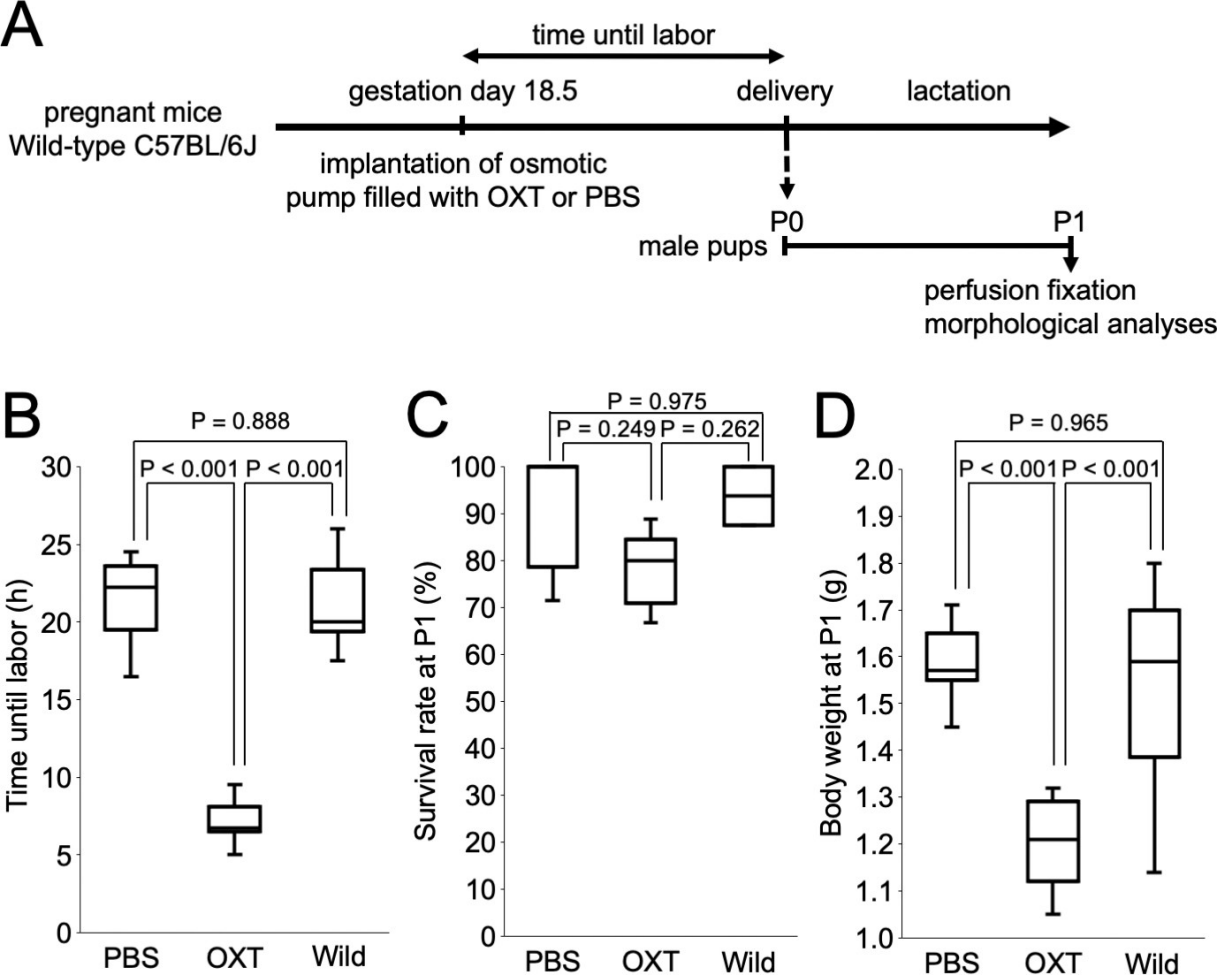
Study design and verification of reproducibility of induced labor model mice. (A) Experimental design of induced labor model and pups. At gestational day 18.5 an osmotic pump was implanted subcutaneously in anesthetized mice. The male pups were analyzed at 24 h (P1) after birth. (B) Time until labor for each group. Time until labor of the oxytocin (OXT) group was significantly shorter than that of the phosphate-buffered saline (PBS) and Wild groups (P < 0.001, Tukey–Kramer method). There were no significant differences between the PBS and Wild groups. (C) Survival rate of OXT, PBS and Wild groups at P1. There were no significant differences between each group. (D) Body weight of OXT, PBS and Wild groups at P1. The body weight of the OXT group was significantly lower than that of the PBS and Wild groups (P < 0.001, Steel–Dwass test). There were no significant differences between the PBS and Wild groups.

We first verified reproducibility of the induced labor model mice (Fig 1A) by measuring the time from implantation of an osmotic pump to delivery of the first pup and survival rates of pups at 24 h (P1) after birth. The labor time was significantly shortened in the OXT group (7.1 ± 0.4 h) compared with the PBS and Wild groups (21.55 ± 0.83 and 21 ± 1.19 h, respectively, P < 0.001, Tukey–Kramer method; Fig 1B). There was no significant difference in the labor time between the PBS and Wild groups (P = 0.888, Tukey–Kramer method). Although some neonates died prior to the experimental endpoint due to maternal neglect, there were no significant differences in the survival rates between the OXT, PBS and Wild groups (78.11 ± 3.63, 91.43 ± 5.71, and 93.75 ± 6.25%, respectively; OXT vs PBS: P = 0.249, OXT vs Wild: P = 0.262, PBS vs Wild: P = 0.975, Steel–Dwass test; Fig 1C). The body weight of the pups was significantly lower in the OXT group (1.21 ± 0.02 g) than in the PBS and Wild groups (1.58 ± 0.02 g and 1.55 ± 0.05 g, respectively; P < 0.001, Steel–Dwass test; Fig 1D) at 24 h after birth, possibly due to earlier delivery in the OXT group. There was no significant difference in body weight between the PBS and Wild groups (P = 0.965, Steel–Dwass test) at 24 h after birth.

### Increased cell death in specific subregions of the PFC and CC in male pups born from induced labor with OXT

For detailed investigation of cell death in the PFC after induced labor, we performed TUNEL and Nissl staining using brain sections from the male neonates 24 h after delivery. In sections corresponding to Figure 61 of the Atlas of the Developing Mouse Brain at E17.5, P0, and P6 [13], we first noticed striking difference in was the forceps minor (FMI) of the corpus callosum (CC). Thus, we decided to perform morphological analysis of FMI of CC besides PFC.

**Fig 2:**
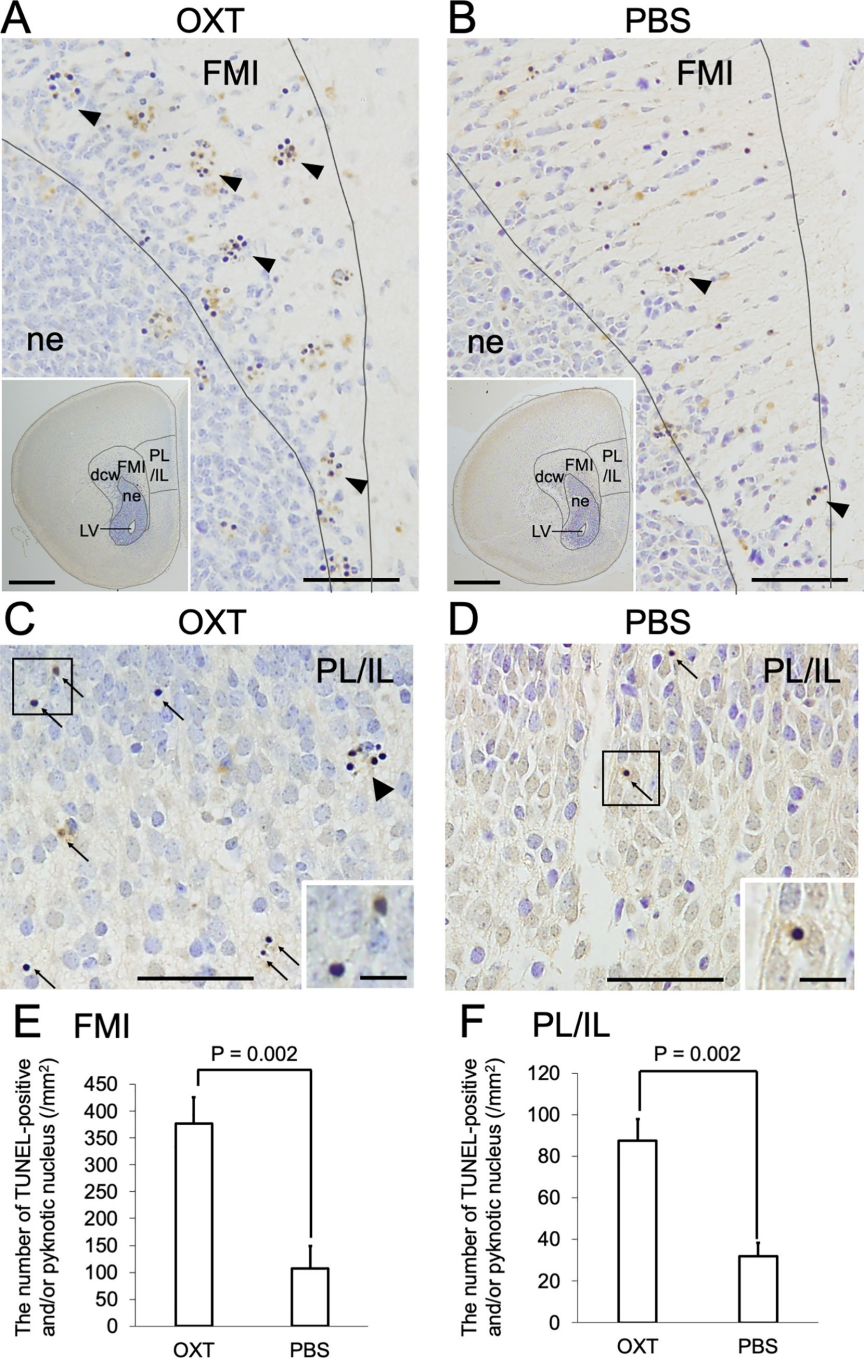
Increased number of pyknotic nuclei and TUNEL-positive cells in forceps minor of corpus callosum and infralimbic and prelimbic subregions of ventromedial prefrontal cortex of OXT group. (A–D) TUNEL- and Nissl-stained sections of OXT (A, C) and PBS (B, D) groups. (A, B) In forceps minor (FMI) of corpus callosum (CC) TUNEL-positive and pyknotic nuclei were more abundant in the OXT group than in the PBS group. TUNEL-positive nuclei were often clustered (arrowheads). The insets are low magnification images of the sections indicating the regions of interest. dcw: deep cerebral white matter, IL: infralimbic cortex, LV: lateral ventricle, ne: neuroepithelium, PL: prelimbic cortex, vmPFC: ventromedial prefrontal cortex. Scale bars: 50 μm and 500 μm (in inset). (C, D) In PL and IL of vmPFC TUNEL-positive and pyknotic nuclei were detected more abundantly in the OXT group than in the PBS group. Squared areas with TUNEL-positive and pyknotic nuclei were enlarged and shown in insets. In the OXT group most of the TUNEL-positive and pyknotic nuclei appeared independently (arrows) rather than as a cluster (bold arrow). Scale bars: 50 μm and 10 μm (in inset). (E) The number of TUNEL-positive and pyknotic nuclei per square millimeter in FMI of CC in the OXT group was significantly larger than that in the PBS group (377.12 ± 48.93 vs 107.04 ± 42.10 for OXT and PBS groups, respectively, P = 0.002, Student’s t-test). (F) The number of TUNEL-positive and pyknotic nuclei per square millimeter in PL and IL was significantly larger than that in the PBS group (87.48 ± 10.57 vs 31.95 ± 6.32 for OXT and PBS groups, respectively, P = 0.002, Student’s t-test).

In the FMI of the CC TUNEL-positive and pyknotic nuclei were more abundant in the OXT group than in that of the PBS group (Fig 2A and 2B). Of note, TUNEL-positive nuclei were primarily found as clusters in the FMI of the OXT group (arrowheads in Fig 2A), whereas in the PBS group TUNEL-positive and pyknotic nuclei appeared either independently or as a cluster (arrowheads in Fig 2B). In infralimbic (IL) and prelimbic (PL) cortex of the ventromedial prefrontal cortex (vmPFC), TUNEL-positive and pyknotic nuclei were also detected more abundantly in the OXT group than in the PBS group (Fig 2C and 2D). In contrast to the FMI of the OXT group, most of the TUNEL-positive and pyknotic nuclei appeared independently (arrows in Fig 2C) rather than as a cluster (bold arrows in Fig 2C). Quantitative analyses confirmed a significant increase in the numbers of TUNEL-positive and pyknotic nuclei in both the FMI of the CC and IL and PL of the vmPFC in the OXT group compared with those in the PBS group (FMI: 377.12 ± 48.93 vs 107.04 ± 42.10/mm^2^, IL and PL: 87.48 ± 10.57 vs 31.95 ± 6.32/mm^2^, respectively, P = 0.002, Student’s t-test) (Fig 2E and 2F). These results suggest that specific subregions of the PFC and CC, namely the FMI of the CC and IL and PL of the vmPFC, were affected in male pups born from an induced labor model with OXT administration. As far as we have observed by Nissl staining, there was no difference in the architecture of IL and PL of the vmPFC between OXT and PBS groups at P40 regardless of the increased cell death in IL and PL of the vmPFC of the OXT group (S1 Fig).

### Enhancement of phagocytosis of dying cells in FMI and vmPFC of male neonatal mice born from induced labor with OXT

**Fig 3:**
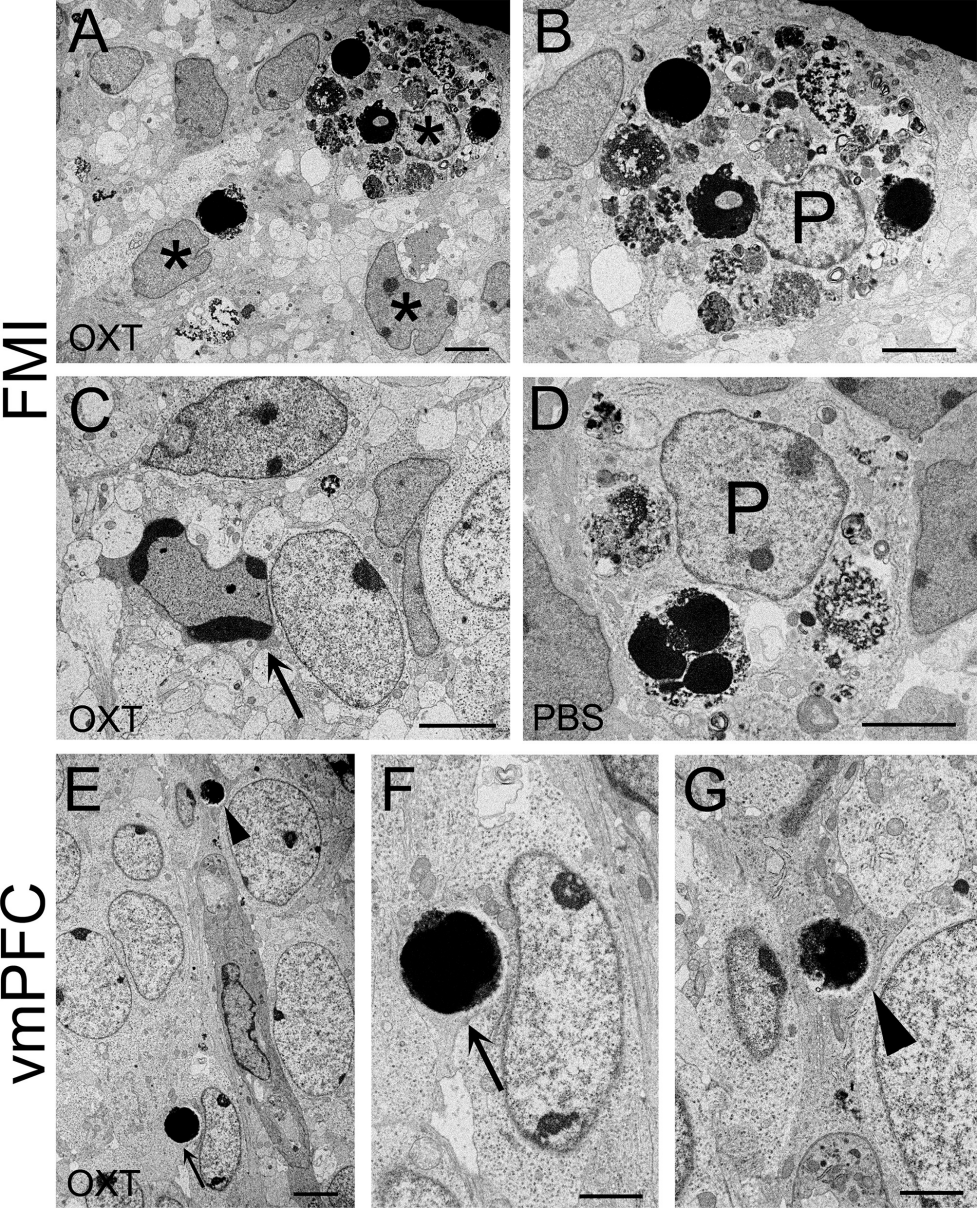
Ultrastructure of dying cells in forceps minor of corpus callosum and ventromedial prefrontal cortex of the male pups at 24 h after delivery. (A–D) Electron micrographs of forceps minor (FMI) of the male pups at 24 h after delivery of the OXT (A–C) and PBS (D) groups. (A) In the OXT group, cells containing pyknotic nuclei and debris of dying cells were abundant (asterisks). (B) An enlarged image of the phagocytic cell (P) shown in A containing many pyknotic nuclei and debris of dying cells. (C) A dying cell with chromatin condensation observed in the OXT group. (D) An enlarged image of the phagocytic cell (P) containing pyknotic nuclei and debris of dying cells in the PBS group. (E–G) Electron micrographs of ventromedial prefrontal cortex (vmPFC) of the male pups at 24 h after delivery of the OXT group. Dying cells with pyknotic nuclei indicated by an arrow and an arrowhead in E were enlarged and shown in F and G, respectively. (F) A dying cell with pyknotic nuclei that was not phagocytosed (arrow). (G) A dying cell with pyknotic nuclei that was encircled by the cytoplasm of the phagocytic cell (arrow). Scale bars: 4 μm (A–E) and 2 μm (F, G).

We observed the ultrastructure of the dying cells at 24 h after delivery by transmission electronic microscopy (Fig 3). Although we observed some cells with chromatin condensation that were not phagocytosed (Fig 3C), most of the dying cells were observed within phagocytic cells, possibly microglial cells, in the FMI of both the PBS and OXT groups (Fig 3A, 3B and 3D). Phagocytic cells also contained debris of dying cells (Fig 3B). Phagocytosed dying cells with pyknotic nuclei were more abundant in the OXT group (Fig 3A and 3B). In the OXT group, phagocytic cells often contained numerous pyknotic nuclei or debris of dying cells, which corresponds with the clusters of pyknotic nuclei observed by Nissl staining (Fig 2A and 2B). In the vmPFC we could find a couple of dying cells with pyknotic nuclei in the OXT group (Fig 3E). Some of the cells were phagocytosed, whereas others were not yet phagocytosed (Fig 3F and 3G). We rarely observed phagocytic cells containing numerous dying cells. This could explain why most of the Nissl-stained pyknotic nuclei appeared independently in the IL and PL of vmPFC (Fig 2C).

### Infiltration of Iba-1-positive microglial cells in IL, PL and FMI of male neonatal mice born from induced labor with OXT

**Fig 4:**
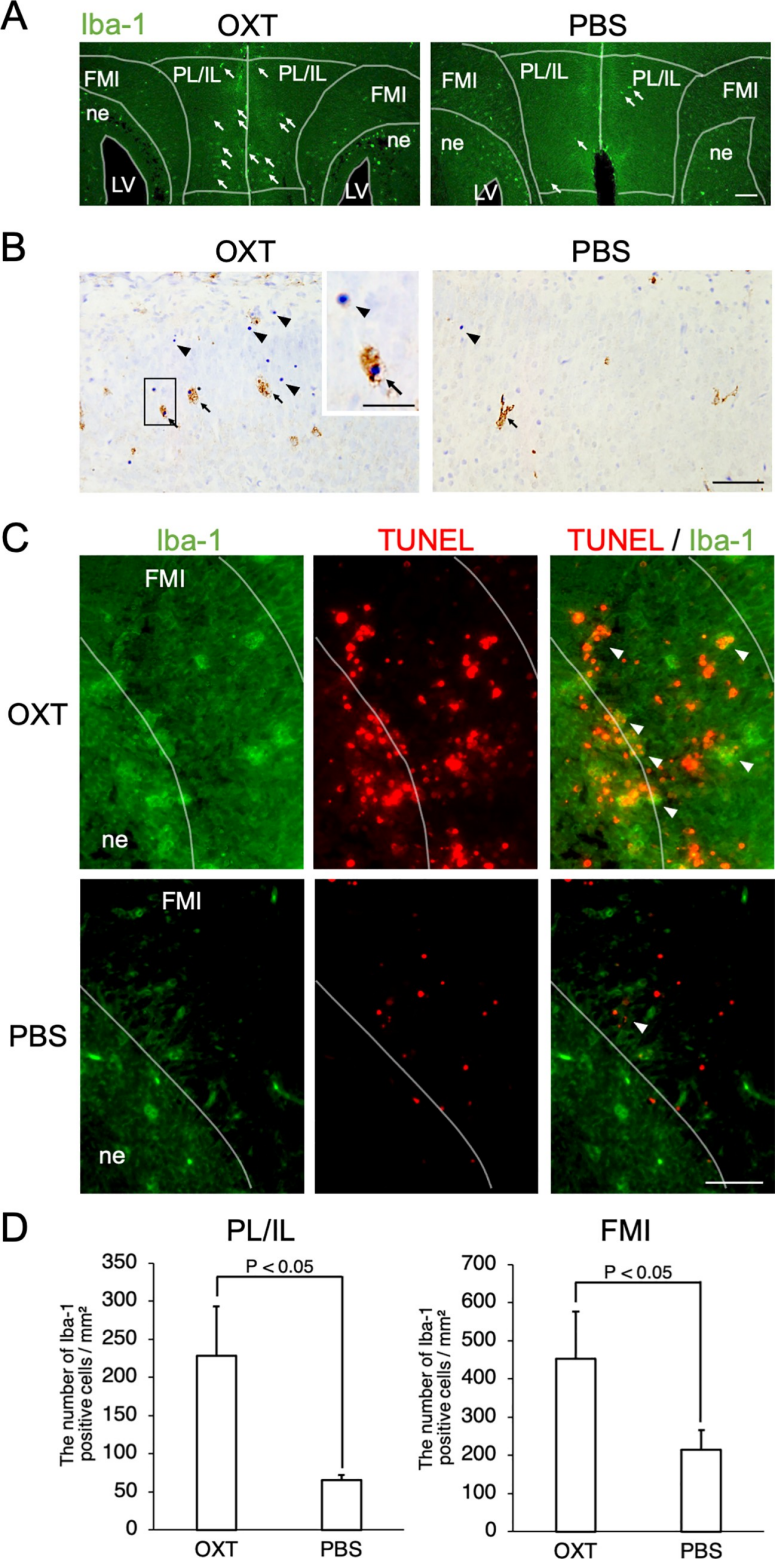
Infiltration of Iba-1-positive microglial cells in forceps minor of corpus callosum and ventromedial prefrontal cortex of the male pups at 24 h after delivery in OXT group. (A) In low power view, the infiltration of Iba-1-positive microglial cells seemed increased in the infralimbic and prelimbic cortex (IL and PL) of the OXT group when compared with that of the PBS group (arrows). FMI: forceps minor, LV: lateral ventricle, ne: neuroepithelium. (B) Immunohistochemistry for Iba-1 in IL of OXT and PBS groups. A squared area was enlarged and shown in the inset. Nissl stain was used for counterstaining. In IL, pyknotic nuclei (arrows) appeared more abundantly and infiltration of Iba-1-positive microglial cells in IL was more pronounced in the OXT group than in the PBS group. In the OXT group some Iba-1-positive microglial cells were engulfing pyknotic nuclei (an arrow in the inset). (C) Images in high power view of the FMI from the same section shown in A. Double labeling for Iba-1 (green), TUNEL staining (red). In FMI, there were more TUNEL-positive nuclei (red) in the OXT group than in the PBS group. In the OXT group many of the Iba-1-positive microglial cells were engulfing clustered TUNEL-positive nuclei (arrowheads). In the PBS group many of the TUNEL-positive cells appeared independently and some Iba-1 positive cells contained TUNEL-positive cells (arrowheads). Scale bars: 100 μm (A) and 50 μm (B, C) and 150 μm (the inset in B). (D) The number of Iba-1-positive microglia per square millimeter in IL and PL in the OXT group was significantly larger than that in the PBS group (228.69 ± 64.71 vs 65.72 ± 5.88 for OXT and PBS groups, respectively, P = 0.016, Student’s t-test). The number of Iba-1-positive microglia per square millimeter in FMI of CC was also significantly larger than that in the PBS group (452.22 ± 124.08 vs 214.34 ± 50.88 for OXT and PBS groups, respectively, P = 0.016, Student’s t-test).

To confirm the transmission electron microscopy observations, we performed double labeling for Iba-1 with either TUNEL or Nissl staining to detect microglial cells and nuclei of dying cells, respectively. In low power view we noticed an increased infiltration of Iba-1-positive microglial cells in the IL and PL of the OXT group when compared with that of the PBS group (Fig 4A). In the IL, microglial cells and TUNEL-positive nuclei appeared more abundantly in the OXT group than in the PBS group (Fig 4B). Some pyknotic nuclei were engulfed with Iba-1-positive microglial cells (an arrow in the inset of Fig 4B). In the FMI, Iba-1-positive microglial cells were detected in both the OXT and PBS groups, whereas there were more TUNEL-positive nuclei in the OXT group than in the PBS group (Fig 4C). In the OXT group, many of the Iba-1-positive microglial cells were engulfing clustered TUNEL-positive nuclei. In the PBS group, many of the TUNEL-positive cells appeared independently and some Iba-1-positive cells contained TUNEL-positive cells (Fig 4C). Quantitative analyses confirmed a significant increase in the numbers of Iba-1-positive microglia in both IL and PL of the vmPFC and the FMI of the CC in the OXT group compared with those in the PBS group (IL and PL: 228.69 ± 64.71 vs 65.72 ± 5.88/mm2, P = 0.008, FMI: 452.22 ± 124.08 vs 214.34 ± 50.88/mm2, P = 0.016, respectively, Student’s t-test) (Fig 4D). These results confirmed that in the subregions of the PFC and CC susceptible to induced labor by OXT administration increased cell death was accompanied by infiltration of microglial cells, which engulfed the dying cells and became activated.

## Discussion

Our previous study showed induced labor with high-dose OXT in pregnant mice resulted brain region-specific cell death in only male offspring [10]. This sex difference of this animal model was assumed to result from the protective effect for brain injury by estrogen and estrogen receptor in female offspring [19, 20]. Brain damage in our model can be explained by hyperactive uterine contractions by OXT rather than pharmacological side effect of OXT itself to neonates, which was supported by the previous result that OXY administration to OXT receptor-knockout mothers did not lead brain damage in the male offspring [10]. Furthermore, OXT itself administered to mothers during induced labor did not influence the expression levels of fetal endogenous OXT and oxytocin receptor [10]. As mentioned in the previous study [10], the concentration of OXT administered to pregnant mice was much higher than is normally administered to humans for induced labor. In human an overdose of OXT may result in uterine hyperstimulation and uterine rupture, both of which may lead to progressive reductions in fetal oxygen levels and be associated with an increased risk of neonatal hypoxic-ischemic brain injury [21, 22]. Therefore, care should be taken when OXT is administered for induced labor.

The present study aimed further detailed morphological investigation of the brain damage in the PFC region in the male offspring of this anima model and provided several insights as follows. First, massive cell death in the perinatal stage of this model was detected in subregions of the vmPFC, especially the IL and PL as revealed by Nissl and TUNEL staining. Besides subregions of the vmPFC we noticed that the FMI of the CC is also severely affected. Third, infiltration of microglial cells engulfing dying cells was pronounced in these regions, as shown by Iba-1 and TUNEL or Nissl staining. These findings strongly suggest the susceptibility of subregions of prefrontal cortex and corpus callosum to damage after induced labor by high-dose OXT in male neonatal mice.

The vmPFC has been implicated in a variety of social, cognitive, and affective functions that are commonly disrupted in several mental disorders [23]. Patients with vmPFC lesions exhibit a sign of social isolation, apathy [24] and decreased prosocial behavior in some social decision-making games [25]. In the present study we found that brain damage occurred in the IL and PL subregions of the vmPFC in male neonatal mice born from induced labor mice with high-dose OXT. The rodent IL and PL regions are homologous to Brodmann areas 25 and 32 of the human vmPFC, respectively [11, 26]. Reduced sociability by isolation rearing differentially alters neural activity in the PL and IL in rats [27]. Structural and functional abnormalities in the PL and IL have been reported in several lines of mice with learning deficits or altered cognitive perception [28, 29]. The FMI of the CC consists of fibers and connects the bilateral PFC, including the pars triangularis, medial PFC, and orbitofrontal cortex [30, 31] which plays a role in language functions [32], and theory of mind and empathy [33] in human, respectively. Diffusion tensor imaging studies have suggested that the FMI is the most implicated in cognitive dysfunctions [34, 35]. Taken together, our results indicate susceptibility within the PFC and CC to damage after induced labor by high-dose OXT in male neonatal mice. As shown above, both regions with brain damage after induced labor have been suggested to be associated with mental disorders in human. As far as we have observed by Nissl staining, there was no difference in the architecture of IL and PL of the vmPFC between OXT and PBS groups at the later stage. Further studies to understand the effect of impairment of these regions in mice would be necessary.

In the present study we examined brain damage by Nissl staining and TUNEL staining because we noticed that the number of the pyknotic nuclei by Nissl staining was much higher than that of the TUNEL-positive nuclei in the damaged brain regions. In the PBS group, we detected several pyknotic nuclei in IL and PL, which indicate programmed cell death. In the cortex, up to 50% of newly differentiated neurons are lost during the establishment of the final number of neurons [36, 37]. In the IL and PL of the OXT group, the number of pyknotic nuclei was greatly increased, indicating damage-induced cell death beyond the programmed cell death. In parallel to the increased detection of pyknotic nuclei, massive infiltration of microglial cells was observed in the OXT group. Microglial cells rapidly migrated to the lesion site, where they phagocytosed dying cells or cellular debris [38]. Our immunohistochemistry confirmed that in the IL and PL, the infiltration of microglial cells selectively occurred in the OXT group and that some of the pyknotic nuclei were already engulfed by microglial cells. In contrast to findings in the IL and PL, microglial cells tended to be detected more frequently in the FMI of CC in both the OXT and PBS groups. However, within these two groups infiltration of microglial cells was more severe in the OXT group, suggesting increased cell death in the FMI of the OXT group compared with that of the PBS group. Furthermore, compared with the microglial cells in the IL and PL, those in the FMI contained many more pyknotic nuclei and debris of dying cells, which was confirmed by immunohistochemistry and electron microscopy. Indeed, most pyknotic nuclei were already engulfed with microglial cells, appearing as a cluster pattern with the Nissl and TUNEL staining.

In the present study we did not identify types of cells vulnerable to induced labor by OXT in the IL, PL and FMI of CC. According to our previous study, oligodendrocyte precursor cells may be one of the candidates [10]. We tried to identify the type of dying cells by electron microscopy or immunohistochemistry. However, the cells with pyknotic nuclei already lacked morphological hallmarks for the identification of cell types by electron microscopy. As mentioned above, many of the dying cells in the IL or PL of the OXT group were being digested by microglial cells. To identify the types of vulnerable cells in our model, a time course analysis would be important to detect the dying cells at an earlier stage after induced labor, before the cells are engulfed by microglial cells. The cluster pattern of pyknotic nuclei was mainly observed in the FMI of the CC but not in the IL and PL. This difference might be due to the difference in the number of dying cells in both regions or in the regional heterogeneity of microglia themselves [39].

Whether microglial phagocytosis is beneficial or harmful in the context of brain injury is a subject of ongoing debate [40]. When brain damage like infection, trauma and ischemia occurs, microglia are rapidly activated, remove exogeneous material and dead cells by phagocytosis, and help to repair tissues by releasing neuroprotective factors [41, 42]. However, excessive activation of microglia can cause neurodegeneration because of the release of a large quantity of inflammatory factors [43, 44]. More recently, microglia have been shown to have important roles besides the above-mentioned immune surveillance and response [45]. Microglia are necessary for synaptic pruning during development [46], for the regulation of the number of functional synapses [47], and for the support of adult neurogenesis [48], indicating that disruption of these microglial functions may contribute to pathological conditions with neuronal or synaptic dysfunction. Notably, dysregulated synaptic development and physiology have been hypothesized to underlie altered neuronal function in several mental disorders [45, 49]. In our animal model, microglial activation might occur as a consequence of increased cell death. Further studies are needed to understand the contribution of pathologically activated microglial cells during the neonatal period to functional and structural abnormalities in the IL and PL of vmPFC and FMI of CC in the later stages of mice born after induced labor by high-dose OXT. Administration of a drug that controls microglial function, such as minocycline [50], is one approach that could be used to investigate this.

In conclusion, we identified the subregions of the PFC and CC vulnerable to brain damage in male neonates after high-dose OXT-induced labor. The identified regions have been associated with mental disorders. Our animal model established in a previous study [10] requires only an osmotic pump implantation procedure and we reproduced our findings of brain damage. As a model of perinatal brain injury, the neonatal hypoxia-ischemia model has been established in rodents and widely used [51, 52]. Along with this, our model would be also useful for assessing the mechanisms of cell death with a milder insult compared to that inducing hypoxia-ischemia in addition to assess the later risk of mental disorders, such as ASD, after induced labor.

## Supporting information

S1 Fig Comparison of architecture of PL and IL of vmPFC between OXT and PBS groups at P40. Nissl-stained sections of OXT (A) and PBS (B) groups. Scale bar: 100 μm.
(TIF)
