# Supplementary figures and images for: Susceptibility of subregions of prefrontal cortex and corpus callosum to damage by high-dose oxytocin-induced labor in male neonatal mice
Source: PLoS One. 2021 Aug 26;16(8):e0256693. doi: 10.1371/journal.pone.0256693 (PMC8389436; doi:10.1371/journal.pone.0256693)

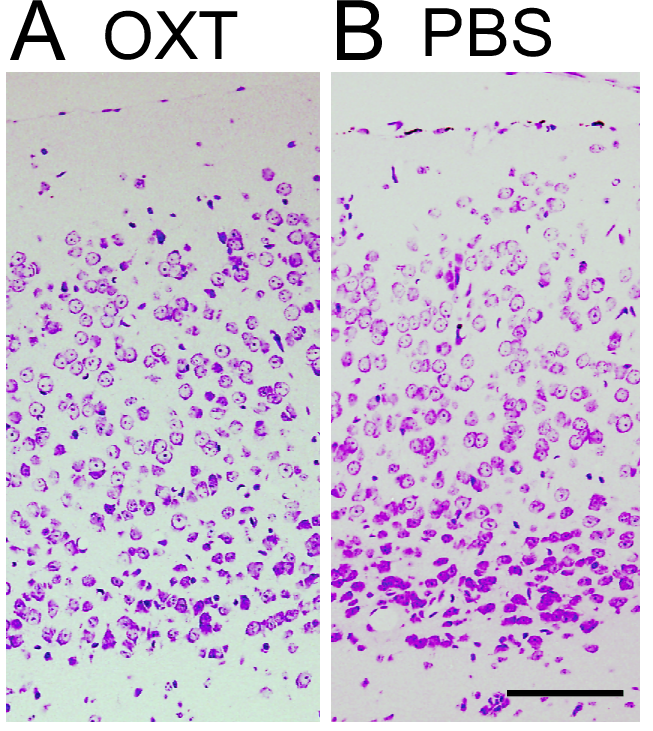

Supplement: S1 Fig — Nissl-stained sections of OXT (A) and PBS (B) groups. Scale bar: 100 μm. (TIF) [file pone.0256693.s001.tif]
